# Multistage Countercurrent Extraction of Abalone Viscera Oil and Its Hypolipidemic Action on High-Fat Diet-Induced Hyperlipidemia Mice

**DOI:** 10.3390/nu17193062

**Published:** 2025-09-25

**Authors:** Meiling Tian, Chunjiang Li, Lili Liu, Fahui Xiang, Weiwei Li, Changcheng Li, Binxiong Liu, Ting Fang

**Affiliations:** 1 College of Food Science, Fujian Agriculture and Forestry University, Fuzhou 350002, China; tml0214@163.com (M.T.); lichunjiang323@163.com (C.L.); liu_lili2025@163.com (L.L.); lww250906@163.com (W.L.); changcheng_li@fafu.edu.cn (C.L.); lbx_zhou@163.com (B.L.); 2 National R&D Center for Vegetable Processing, Fuzhou 350002, China; 3 Fujian Key Laboratory of Agro-Products Quality & Safety, Institute of Quality Standards and Testing Technology for Agro-Products, Fujian Academy of Agricultural Sciences, Fuzhou 350003, China; xiangfahui08@163.com

**Keywords:** abalone viscera oil, multistage countercurrent extraction, antioxidant, fatty acid composition, hypolipidemic action

## Abstract

Background/Objectives: Marine-derived oils rich in long-chain polyunsaturated fats have long been associated with positive effects on plasma lipid levels and anti-inflammatory responses. Abalone viscera are rich in oils that are rarely extracted and made available. Methods: Abalone viscera oil (AVO) was extracted by multistage countercurrent extraction using ethanol as a solvent, and its oil quality, fatty acid composition, and in vitro antioxidant activity were determined. Meanwhile, the anti-hyperlipidemic effect of AVO on HFD-induced hyperlipidemia mice was evaluated. Results: The abalone viscera were extracted at a solid–liquid ratio of 1:3 with an oscillation frequency of 300 rpm for 40 min, and the extraction rate was 81.18% after four-stage countercurrent extraction. The acid value, iodine value, peroxide value, vitamin E, and astaxanthin of AVO were 1.26 mg KOH/g, 140.9 g/100 g, 3.6 meq/kg, 105 mg/kg, and 533.8 mg/kg, respectively. The unsaturated fatty acids of AVO account for 56.60%, with eicosapentaenoic acid (C20:5n3) and arachidonic acid (C20:4n6) the two predominant PUFAs, and oleic acid (C18:1n9) the most dominant MUFA. The DPPH, ABTS, and **·**OH radicals scavenging capacities of AVO increased with concentration, and the IC_50_ values were 6.30 mg/mL, 0.45 mg/mL, and 8.95 mg/mL, respectively. Moreover, the administration of AVO significantly alleviated HFD-induced weight gain, liver fat accumulation, lipid disorder, and oxidative stress in mice. Conclusions: Collectively, our study provides a theoretical basis for the application of AVO and the comprehensive utilization of abalone viscera, which helps increase the additional value of abalone.

## 1. Introduction

Marine oils extracted from deep-sea fish, shrimp, shellfish, krill, microalgae, and other large marine animals are rich in bioactive compounds, especially polyunsaturated fatty acids (PUFAs), such as omega-3 and omega-6 fatty acids [1]. Prospective observational studies support the role of long-chain omega-3 polyunsaturated fatty acids eicosapentaenoic acid (EPA, C20:5n3) and docosahexaenoic acid (DHA, C22:6n3) in the primary prevention of atherosclerotic cardiovascular disease [2,3]. Moreover, long-chain omega-3 PUFAs supplementation can reduce the risk of death from myocardial infarction, coronary heart disease, and atherosclerotic cardiovascular disease in participants, and the reduction in mortality risk is linearly correlated with intake [2]. Substituting saturated fatty acids (SFAs) with omega-6 PUFAs can help reduce the risk of myocardial infarction in human beings, but there is no dose dependency [2]. Marine oils significantly reduce the risk of developing atherosclerotic cardiovascular disease events by controlling dyslipidemia, a primary risk factor for such conditions [4]. Kim et al. found that krill oil supplements significantly reduced triglyceride (TG), total cholesterol (TC), and low-density lipoprotein cholesterol (LDL-C) levels in the liver and serum of hypercholesterolemic rats, thereby lowering the risk of atherosclerosis [5]. Eel oil, cod liver oil, and trout oil significantly reduce blood glucose and cholesterol levels in mice with hyperlipidemia induced by a high-fat diet [6]. Dietary fish oil significantly reduced serum TC, TG, and non-HDL cholesterol levels in patients with hyperlipidemia [7]. Therefore, marine oils rich in UFAs are promising functional food ingredients in alleviating hyperlipidemia.

Marine organism oils can be extracted using various methods, including organic solvent extraction, enzymatic hydrolysis, and supercritical CO_2_ fluid extraction. The solvent method is one of the conventional methods for extracting oil from marine organisms, with hexane, petroleum ether, and ethanol being commonly used organic solvents [8]. This method is easy to operate and low in cost, but it has a relatively low extraction rate and produces a large amount of residual solvent [8]. Enzymatic hydrolysis utilizes proteases to break down the bonds between proteins and fats, offering the advantages of mild reaction conditions, high extraction efficiency, and environmental friendliness [9]. However, the enzymatic reaction time is relatively long, and the oil yield is relatively low. Supercritical CO_2_ fluid extraction is an emerging solvent extraction technology that offers advantages such as moderate temperatures, an oxygen-free environment, and low contaminant content [8]. Its main drawback is the high cost of industrial-scale application.

Multistage countercurrent extraction is a separation process that achieves efficient separation by repeatedly exposing the components of a mixture to a solvent in countercurrent flow [10]. With the same amount of solvents used, the countercurrent configuration significantly improves separation efficiency and achieves the highest extraction efficiency. Multiple studies have reported the application of multistage countercurrent extraction technology for isolating various bioactive compounds, such as glycyrrhizic acid from *Glycyrrhiza uralensis* Fisch, antioxidants from *Ginkgo biloba* L. leaves, and protein from *Jatropha curcas* seeds [11,12,13]. For oil, Bessa et al. used a multistage countercurrent extraction method with ethanol as the solvent to extract oil from rice bran [10]. After five complete cycles, the residual oil content in the final extract was less than 0.5%, and the fatty acid composition of the oil was typical of rice bran oil. Ferreira et al. used the same method to extract soybean oil and found that after five stages of extraction, only 0.17% of oil remained in the solid phase, with an extraction rate of 99.2% [14]. Moreover, substituting ethanol for hexane, the primary solvent in oil extraction, offers a safer, more eco-friendly solution with significant cost-effectiveness [10,14]. However, to date, no information has been published regarding the extraction of abalone viscera oil using multistage countercurrent extraction technology.

China is the largest producer and consumer of farmed abalone (*genus Abalone*, *family Abaloneidae*, *order Archaeopteryx*), with the production amounting to 24.50 × 10^4^ tonnes in 2023 [15]. Nevertheless, the abalone viscera, which account for approximately 25% of the total weight of abalone, are currently mostly processed into feed or discarded directly and have not yet been effectively utilized [16,17]. Abalone viscera are rich in nutritional ingredients such as protein, polysaccharides, and oils. Polysaccharides extracted from the viscera of abalone (*Haliotis discus hannai Ino*) exhibited dose-dependent and time-dependent anti-tumor activity against gastric carcinoma cells via the apoptosis pathway [18]. Additionally, abalone visceral polysaccharides have remarkable lipid-lowering and anti-atherosclerotic activities [19]. Peptides extracted from abalone viscera using trypsin showed significant angiotensin-converting enzyme inhibitory activity [20]. Recent studies have demonstrated that collagen peptides derived from abalone viscera exhibit anti-inflammatory properties, which may be achieved by regulating the gut microbiota and alleviating oxidative stress [17]. However, compared to the peptides and polysaccharides in abalone viscera, research on the extraction and functional evaluation of abalone viscera lipids is relatively scarce.

To address the significant waste of abalone viscera, in-depth development of this resource and exploration of high-value utilization pathways should be pursued. Moreover, to enhance the extraction efficiency, a multistage countercurrent extraction process using ethanol as a solvent was employed to extract oil from abalone viscera. The quality, fatty acid composition, vitamin E and astaxanthin contents, and in vitro antioxidant activity of abalone viscera oil (AVO) were determined. We also evaluated the anti-hyperlipidemic effect of AVO on HFD-induced hyperlipidemia in mice. This research provides innovative solutions for the high-value utilization of abalone viscera, which not only solves the problem of processing by-product waste but also develops new ingredients for functional food additives and dietary supplements.

## 2. Materials and Methods

### 2.1. Preparation of AVO

The abalone (*Haliotis discus hannai*) viscera, which were kindly provided by Zhaoan Hailian Food Co., Ltd. (Zhangzhou, China), were dried by vacuum freeze-drying, crushed in a pulverizer, and sieved through a 60 mesh to achieve the final product labeled abalone viscera powder, which contained 20.03% oil determined by Soxhlet extraction. The crude AVO (cAVO) was extracted by a multistage countercurrent extraction process using ethanol as solvent. The number of ideal stages for the extraction was calculated according to the description of our previous study, and four stages were obtained [21]. The yield of cAVO was used as an indicator to evaluate the extraction efficiency. Different solid–liquid ratio (the rate of abalone viscera powder and ethanol, 1:2, 1:3, 1:4, 1:5, and 1:6), extraction time (20, 30, 40, 50, and 60 min), oscillation frequency (100, 150, 200, 250, and 300 rpm), and ethanol ratio in water (70%, 80%, 90%, 95%, and 100%) were used to select single factor ranges for orthogonal experiment. An orthogonal design trial L9 (3^3^) was used to optimize the extraction procedure. cAVO was further refined following the previous study to obtain the final product of AVO [21].

### 2.2. Extraction Rate Determination

The extraction rate of cAVO was determined by the Soxhlet extraction method. Briefly, 5 g of abalone viscera powder was placed in a Soxhlet extractor, and 300 mL of ethanol was added to the receiver bottle. The receiver bottle was placed in a water bath at 85 °C, and the extraction was carried out for 6 h. The ethanol-grease mixture was concentrated to dryness in a rotary vacuum evaporator (RE-3000, Yarong Biochemical Instrument, Shanghai, China) at 85 °C and then dried in an electro-thermostatic blast oven (Shanghai Yiheng Scientific Instrument Co., Ltd., Shanghai, China) at 101 °C until constant weight. cAVO extraction rate was analyzed as follows.

(1) $$cAVO\;extration\;rate\;\left(\%\right)=\frac{Total\;yield\;(g)\;extracted\;by\;multistage\;countercurrent\;extraction}{Total\;yield\;(g)\;extracted\;by\;Soxhlet\;extraction}\times 100$$

### 2.3. Acid Value, Iodine Value, and Peroxide Value Determination

Acid value, iodine value, and peroxide value of AVO were tested according to the method described in GB 5009.229-2016 [22], GB/T 5532-2022 [23], and GB 5009.227-2023 [24].

### 2.4. Fatty Acid Compounds Analysis

Fatty acid of AVO (0.5 g) was extracted using 2 mL of methanol solution containing 20% (*v*/*v*) boron trifluoride and 2 mol/L of KOH. The sample was vortexed for 1 min, incubated in a water bath at 75 °C for 15 min, cooled with running water, and 1.5 mL of methanol solution containing 3 mol/L hydrochloric acid was added. The mixture was then blended, incubated in a 75 °C water bath for 15 min again, and cooled with running water. The mixture was then added with 2 mL of n-hexane and 5 mL of deionized water, shaken thoroughly, left to stratify, and the upper organic layer was obtained. The upper solution was then filtered using a 0.2 µm nylon syringe filter for further analysis.

The sample was analyzed using an Agilent gas chromatography–mass spectrometry (5975C-7890A, Agilent Technologies, Santa Clara, CA, USA) using a capillary Agilent 112-88A7 HP-88 (100 m× 0.25 mm i.d. × 0.20 μm) column. Nitrogen (99.999%) was used as the carrier gas at a flow rate of 1.5 mL/min. The temperature program was set at 145 °C for 25 min, increased by 3 °C/s to 230 °C, and maintained for 10 min. The temperature of the injector was 250 °C, and the split ratio was 20:1. Data were collected using Chromeleon Chromatography Studio software (version 7.1.0.898, Dionex Corporation, Sunnyvale, CA, USA). The contents of individual fatty acids were given as a percentage of all fatty acids detected.

### 2.5. Vitamin E Analysis

Vitamin E content in AVO was measured according to a previous study with some modifications [25]. In brief, 5 g of AVO, 30 mL of ethanol, 5 mL of 10% vitamin C, and 20 mL of 1 mol/L NaOH were sequentially added to a saponification flask and saponified in a boiling water bath for 1 h. The mixture was then supplemented with 50 mL of ether, vortexed thoroughly for 60 s, and left to stratify. The upper layer was extracted twice with 50 mL of ether in a separatory funnel and washed to neutrality with deionized water, evaporated to dryness under a gentle stream of nitrogen. The dried sample was dissolved in methanol and filtered through a 0.22 μm nylon syringe filter.

The resulting supernatant was analyzed on a Waters Acquity UPLC system (Waters, Milford, MA, USA) equipped with a photo diode array detector with Acquity BEH C18 (2.1 mm × 100 mm, 1.7 μm). The detection was conducted with an injection volume of 2.0 μL, an isocratic mobile phase of methanol-water (92:8, *v*/*v*), a flow rate of 0.3 mL/min, a column temperature of 35 °C, and a detection wavelength of 290 nm. Vitamin E concentration was calculated based on the peak area of standard samples (Sigma-Aldrich, St. Louis, MO, USA).

### 2.6. Astaxanthin Analysis

Astaxanthin of AVO (0.5 g) was extracted with 30 mL of acetonitrile containing 10 g of sodium sulfate anhydrous. The sample was mixed, sonicated for 10 min, and centrifuged at 3000× *g* for 5 min to obtain the supernatant. The supernatant was extracted twice with 20 mL of n-hexane in a separatory funnel, added with 5 mL of n-propanol, and evaporated to dryness under a gentle stream of nitrogen. The dried sample was dissolved in 5 mL of acetonitrile and filtered through a 0.22 μm nylon syringe filter.

The resulting supernatant was analyzed on an Agilent 1200 HPLC system (Agilent Technologies, Santa Rosa, CA, USA) equipped with a diode array detector and an Agilent C18 column (4.6 mm × 150 mm, 5 μm). The detection was conducted with an injection volume of 50 μL, an isocratic mobile phase of acetonitrile-water (95:5, *v*/*v*), a column temperature of 35 °C, a flow rate of 1.0 mL/min, and a detection wavelength of 471 nm. The concentration of astaxanthin was calculated from the peak area of standard samples (Sigma-Aldrich, St. Louis, MO, USA). 

### 2.7. In Vitro Antioxidant Activities

AVO samples were dissolved in absolute ethanol to prepare sample solutions with final concentrations of 0.2, 0.4, 0.6, 0.8, 1.0, 3.0, 5.0, 7.0, and 9.0 mg/mL, respectively. DPPH, ABTS, and **·**OH free radical scavenging capacity of AVO at different concentrations and VE at 0.125 mg/mL (positive control) were measured following the manufacturer’s instructions (Solarbio Co., Ltd., Beijing, China).

### 2.8. Animal Experiments

Forty male specific-pathogen-free (SPF) Kunming (KM) mice (7 weeks, 30–35 g) were purchased from Huafukang Biotechnology Co., Ltd. (Beijing, China) (Animal Certificate Number: SCXK (Jing) 2019-0008). The mice were housed in 10 cages (4 mice per cage) at the SPF animal facility with a temperature of 24 ± 1 °C and a 12/12 h light-dark cycle, and had ad libitum access to water and food. Regular testing of microbial contamination and pressure gradients is performed to ensure the integrity of the barrier system. The sample size calculation was performed by G*Power software (version 3.1.9.2, Universität Kiel, Kiel, Germany) using an F-test (ANOVA: fixed effects, omnibus, one way) with an effect size of 0.6, a significance level of 95% and a power of 80%, giving a total sample size of 40 individuals (8 in each group). The animal experiments were carried out following the protocol approved by the Animal Care and Use Committee of Fujian Agriculture and Forestry University (Approval number: PZCASFAFU23044. Date: 9 March 2023).

After one week of acclimatization, the mice were randomly divided into a normal diet group (NC = 8) and a high-fat diet group (HD, n = 32). The mice in the NC group were fed a normal diet (H10010, Huafukang Biotechnology Co., Ltd., Beijing, China) for the entire experiment, while mice in the HD group were fed a high-fat diet (H10045, Huafukang Biotechnology Co., Ltd., Beijing, China). The composition of the normal chow and high-fat diet is listed in Table S1. The body weight and serum TG and TC levels of mice fed a high-fat diet for 14 days were significantly higher than those of the NC group, indicating that the hyperlipidemia model had been successfully established (Table S2). The mice in the HD group were then further divided into HFD, LAVO, MAVO, and HAVO groups, where the mice were fed a high-fat diet and gavaged with 0, 250, 500, and 1000 mg/kg body weight of AVO with cholesterol-free Mazola corn oil as vehicle, respectively, for 4 weeks. Mice in the control group were gavaged with the same volume of corn oil as the four experimental groups. The dose was calculated by converting the daily intake for adults (2 g, 4 g, and 8 g correspond to low, medium, and high doses, respectively) to the daily intake for mice. The body weight and emotional state of the mice were recorded in the morning of each day to monitor the changes in body weight and the experimental endpoint, respectively.

At the end of the experiment, the mice were anesthetized with ether after an 8-h fast. The blood samples were collected from the posterior orbital venous plexus, incubated at room temperature, and then centrifuged at 1500× *g* for 10 min to obtain serum. Subsequently, the mice were euthanized by cervical dislocation, and the livers, kidneys, and hearts were collected and weighed. Additionally, livers were fixed with 10% formalin and used for HE staining and blind assessment. The organ index (%) was calculated as: organ weight (g)/body weight at sacrifice (g) × 100%.

### 2.9. Determination of Biochemical Indicators in Serum

Serum biochemical indicators were measured using commercial kits (Nanjing Jiancheng Institute of Bioengineering, Nanjing, China) of TG (A110-1-1), TC (A111-1-1), high-density lipoprotein cholesterol (HDL-C, A112-1-1), and LDL-C (A113-1-1), malondialdehyde (MDA, A003-1-1), nitric oxide (NO, A013-2-1), glutathione peroxidase (GSH-Px, A005-1-2), and total superoxide dismutase (T-SOD, A001-1-2), tested according to the manufacturer’s instructions. Atherogenic coefficient (AC) was calculated as follows: AC = (TC − HDL-C)/HDL-C [26].

### 2.10. Data Analysis

Data are expressed as mean ± SD. The difference between groups was processed with the SPSS 20.0 software (IBM Corporation, Armonk, NY, USA) using a one-way analysis of variance (ANOVA) followed by Duncan’s multiple range test (equal variances assumed) or Tamhane’s T2 test (equal variances not assumed). The power value was calculated by the SPSSAU online software (https://spssau.com/indexs.html, accessed on 19 September 2025). The difference between groups was considered to be significant at *p* < 0.05. Unless otherwise stated, figures were plotted using GraphPad Prism software (7.0 version, GraphPad Software, La Jolla, CA, USA).

## 3. Results

### 3.1. Optimization of the cAVO Extraction Process

**Figure 1:**
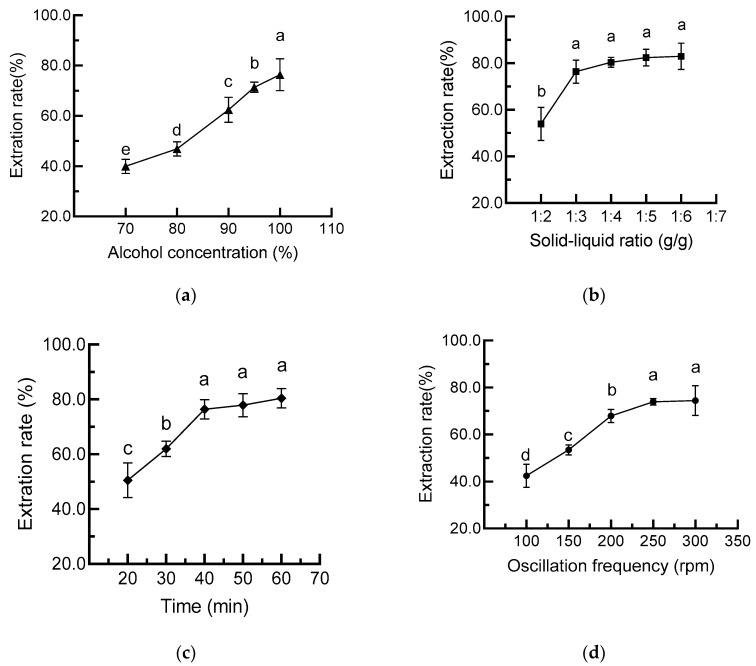
Effects of ethanol concentration (**a**), solid–liquid ratio (**b**), extraction time (**c**), and oscillation frequency (**d**) on cAVO extraction rate. cAVO, crude abalone viscera oil. Different letters indicate statistical significance (*p* < 0.05) between different treatments.

The effects of the ethanol ratio in water on cAVO extraction rate were first evaluated. As shown in Figure 1a, the extraction rate of cAVO increased with increasing ethanol concentration. The highest cAVO extraction rate of 15.3% was obtained at an ethanol concentration of 100%. Consistently, Bessa et al. used anhydrous ethanol to extract rice bran oil through a multistage countercurrent extraction method and demonstrated that the ethanol extraction method is a promising alternative to traditional extraction methods [10]. Therefore, anhydrous ethanol was chosen as the extraction solvent for this study.

The influences of solid–liquid ratio, extraction time, and oscillation frequency on cAVO extraction rate were further evaluated to select a range of extraction parameters for orthogonal experiments (Figure 1b–d). The results showed that the extraction rate of cAVO increased with the solid–liquid ratio, extraction time, and oscillation frequency. The extraction rate was no longer significantly improved when the solids-to-liquid ratio, extraction time, and oscillation frequency were set to 1:4, 40 min, and 250 rpm, respectively. Considering the cost issue, 1:2, 1:3, and 1:4 of solids to liquid ratio; 30 min, 40 min, and 50 min of extraction time; 200 rpm, 250 rpm, and 300 rpm of oscillation frequency, were selected for the orthogonal design trial L9 (3^3^). The R values for factors A, B, and C were 0.91, 1.06, and 0.37, respectively (Table 1), which suggests that the extraction time (factor B) had the highest influence on the extraction rate of cAVO, followed by the solid–liquid ratio (factor A) and oscillation frequency (factor C). The optimal process combination was B_2_A_2_C_3_, indicating that the highest extraction rate of cAVO was achieved under conditions of a solid–liquid ratio of 1:3, an oscillation frequency of 300 rpm, and a processing time of 40 min.

The influence of each factor on the experimental results was further analyzed and presented in Table 2. The F values of factor A and factor B were between F_0.05_ (2, 2) and F_0.01_ (2, 2), which indicated that factors A and B, namely solid–liquid ratio and extraction time, had a significant effect on the extraction rate of cAVO. The F value of factor C was less than F_0.05_ (2, 2), indicating that the oscillation frequency had no significant impact on the cAVO extraction rate. Therefore, we chose a solid–liquid ratio of 1:3, an oscillation frequency of 250 rpm, and an oscillation time of 40 min for the extraction of cAVO. The cAVO was further refined to obtain the AVO.

### 3.2. AVO Quality

To evaluate the quality of AVO, the acid value, iodine value, and peroxide value were determined and shown in Table 3. The acid value and peroxide value of AVO were 17.25 and 1.89-fold lower than cAVO, and the iodine value increased from 123.6 g/100 g to 140.9 g/100 g after refining. All these indices had reached the aquatic industry standard in China and Codex Alimentarius Commission standard for fish oil after the refining [27,28], indicating that high-quality AVO was obtained.

**Figure 2:**
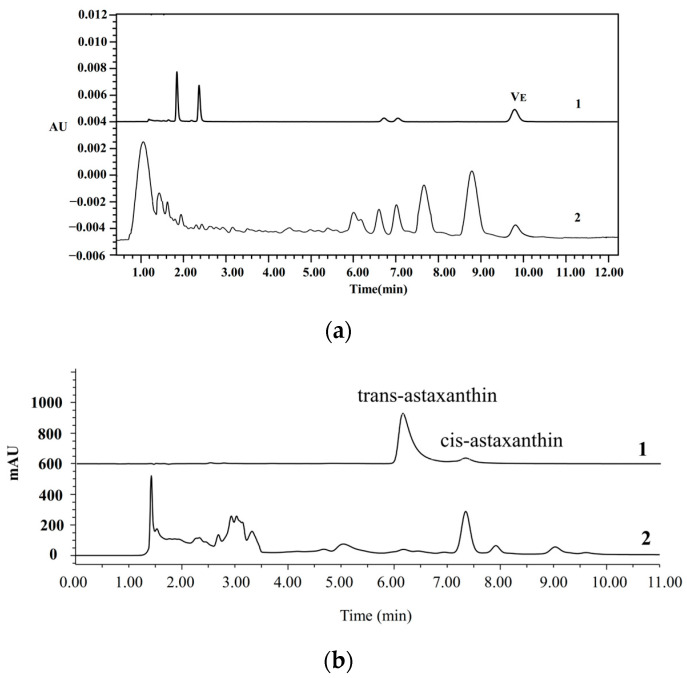
Representative HPLC chromatograms of vitamin E (**a**) and astaxanthin (**b**) in AVO. 1 and 2 represent the chromatograms of the standard and AVO sample, respectively.

Vitamin E, a fat-soluble vitamin, is a crucial micronutrient in oil and serves as an indicator of oil quality. The content of vitamin E of AVO was 105 mg/kg (Figure 2a), which was comparable to the vitamin E content of fish (*Coregonus peled*) fat oil determined by HPLC in a previous study [29]. However, the content is lower than that found in 33 dietary oil supplements available on the German market (such as fish oil, krill oil, and microalgae oil), which range from 1.2 to 86.1 mg/g [30]. Astaxanthin, a major keto-carotenoid isolated from crustaceans and salmon, is one of the world’s most powerful natural antioxidants and exists in both cis and trans forms [31]. The total astaxanthin content of AVO was 533.8 ± 2.6 mg/kg, with cis-astaxanthin content being much higher than the trans one (Figure 2b). The astaxanthin content of AVO was higher than the astaxanthin level of 21.81 µg/g detected in salmon frame bone oil [32]. Moreover, the content of astaxanthin is comparable to 440–690 mg/kg in krill oil extracted by the Folch and subcritical butane-dimethyl method, while being lower than 910 mg/kg and 940 mg/kg using the ethanol–hexane method and the subcritical butane method, respectively [33]. This indicates that the AVO obtained in this study exhibits favorable quality. 

### 3.3. Fatty Acid Composition of AVO

The fatty acid composition of AVO is presented in Table 4, and the results indicate that 23 fatty acids are present in AVO. These fatty acids consisted of 8 SFAs, 7 MUFAs, and 8 PUFAs with a ratio of 2.17:1:1.83. Furthermore, palmitic acid (C16:0) and myristic acid (C14:0) were the most abundant SFA occurring in AVO, oleic acid (C18:1) was the most abundant MUFA, and eicosatetraenoic acid (C20:4) and EPA (C20:5n3) were the most abundant PUFA. These results were similar to the fatty acid profile of abalone viscera oil extracted previously using the three-liquid-phase salting-out extraction system (n-hexane/ethanol/sodium citrate/citric acid) [34]. The UFAs content was higher than the SFA, and the most abundant fatty acids were palmitic acid (C16:0), myristic acid (C14:0), oleic acid (C18:1n9), arachidonic acid (ARA, C20:4n6), and EPA (C20:5n3) [34]. These results indicate that the AVO extracted by the multistage countercurrent extraction method used in this study can maintain the unique fatty acid profile of abalone oil.

### 3.4. Antioxidant Capacity of AVO In Vitro

**Figure 3:**
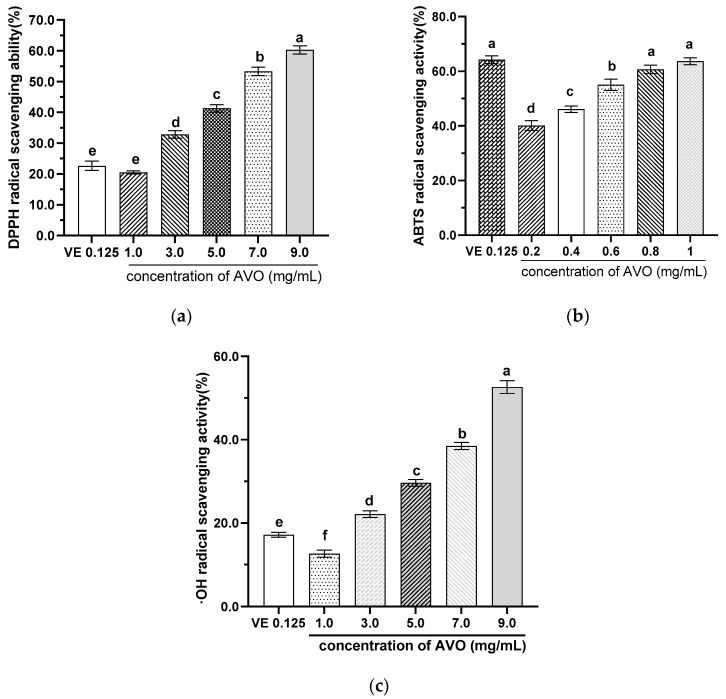
Scavenging capacities of AVO on DPPH (**a**), ABTS (**b**), and **·**OH (**c**) radicals. AVO, abalone viscera oil. Different letters indicate statistical significance (*p* < 0.05) between groups.

As described above, AVO has a higher content of UFAs than SFAs and contains a certain amount of Vitamin E and astaxanthin, indicating that AVO has potential antioxidant properties. Therefore, the in vitro antioxidant capacity of AVO was evaluated. The results demonstrated that the DPPH, ABTS, and **·**OH radicals scavenging capacities of AVO were increased with concentration, and the IC_50_ values were 6.30 mg/mL, 0.45 mg/mL, and 8.95 mg/mL, respectively (Figure 3). The IC_50_ value of DPPH and **·**OH were higher than 0.148 mg/mL of fish liver oil and 4.13–2.07 mg/mL of Klunzinger’s mullet (*Liza klunzingeri*) muscle hydrolysates, respectively [35]. However, the IC_50_ of ABST was comparable to cod liver oil, which showed 50.07% inhibition of ABTS free radical scavenging activity at a concentration of 0.5 mg/mL [35]. These results suggest that AVO exhibits certain in vitro antioxidant properties.

### 3.5. Anti-Hyperlipidemic Effects of AVO In Vivo

#### 3.5.1. Effect of AVO on the Body Weight, Organ Index, and Liver Histopathology of Mice

**Figure 4:**
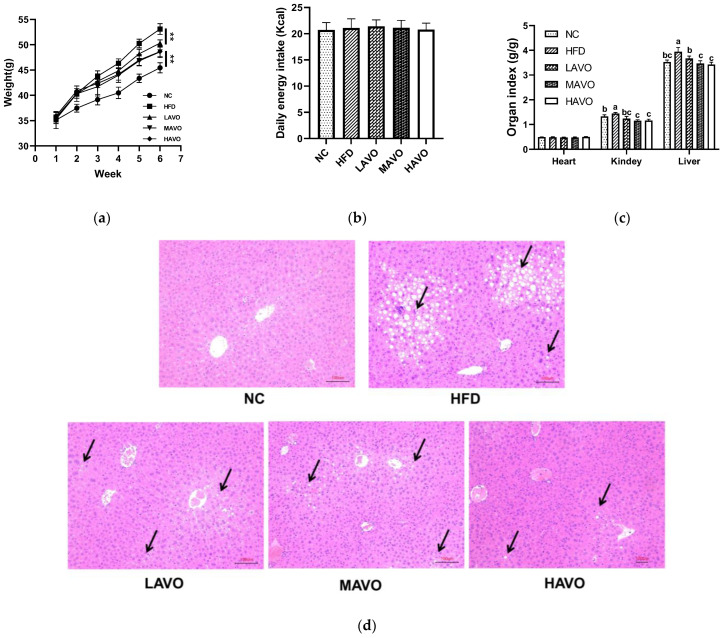
Effect of AVO on the body weight (**a**), daily energy intake (**b**), organ index (**c**), and histopathological examination of liver sections (**d**) of HFD-induced mice (n = 6). The black arrow represents the accumulation of lipid droplets. AVO, abalone viscera oil; NC, normal control group; HFD, high-fat diet group; LAVO, high-fat diet + 250 mg/kg AVO; MAVO, high-fat diet + 500 mg/kg AVO; HAVO, high-fat diet + 1000 mg/kg AVO. Different letters indicate statistical significance (*p* < 0.05) between groups. ** *p* < 0.01.

The protective effects of AVO against hyperlipidemia were evaluated in mice fed with HFD. The results showed that AVO administration significantly reduced body weight gain by 5.24–8.50%, without altering the daily energy intake (Figure 4a,b). The kidney and liver index in HFD mice were also reduced considerably after AVO supplementation, while the heart index showed no significant change (Figure 4c). Moreover, the H&E staining of liver sections showed a remarkable accumulation of lipid droplets in the HFD group, whereas the hepatic steatosis was significantly improved by AVO intervention at different doses (Figure 4d). These results indicated that supplementation with AVO significantly alleviated fat accumulation.

#### 3.5.2. Effect of AVO on Serum Lipid Levels and AC in Mice

**Figure 5:**
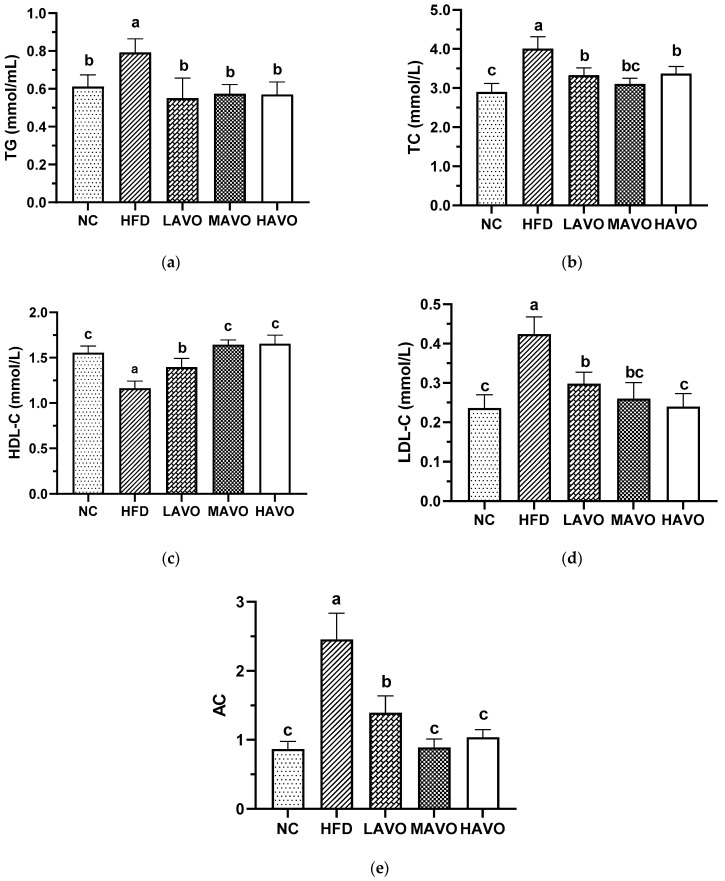
Effect of AVO on TG (**a**), TC (**b**), HDL-C (**c**), and LDL-C (**d**) levels and AC (**e**) in mouse serum (n = 6). AVO, abalone viscera oil; NC, normal control group; HFD, high-fat diet group; LAVO, high-fat diet + 250 mg/kg AVO; MAVO, high-fat diet + 500 mg/kg AVO; HAVO, high-fat diet + 1000 mg/kg AVO. Different letters indicate statistical significance (*p* < 0.05) between groups.

Hyperlipidemia is characterized by low serum HDL-C levels and high serum TC, TG, and LDL-C levels [36]. The AC, also known as the atherosclerosis burden or the atherosclerotic index of plasma, is a metric that reflects the degree of risk for cardiovascular and cerebrovascular diseases [26]. In our study, serum levels of TG, TC, LDL-C, and AC were significantly elevated by 29.41%, 38.11%, 79.66%, and 103.10% in HFD-fed mice compared to the ND group, respectively, while HDL-C levels significantly decreased by 31.96% (Figure 5). AVO administration significantly reversed the HFD-induced decrease in serum HDL-C levels and reduced the HFD-induced increases in serum TG, TC, LDL-C, and AC levels in mice (Figure 5). These findings are consistent with previous studies demonstrating the effects of edible oils rich in PUFAs on lipid level changes in HFD-induced obese mice [37,38]. Collectively, our results indicate that dietary supplementation with AVO significantly improves lipid abnormalities induced by HFD.

#### 3.5.3. Effect of AVO on Oxidative Stress Factors in Mice Serum

**Figure 6:**
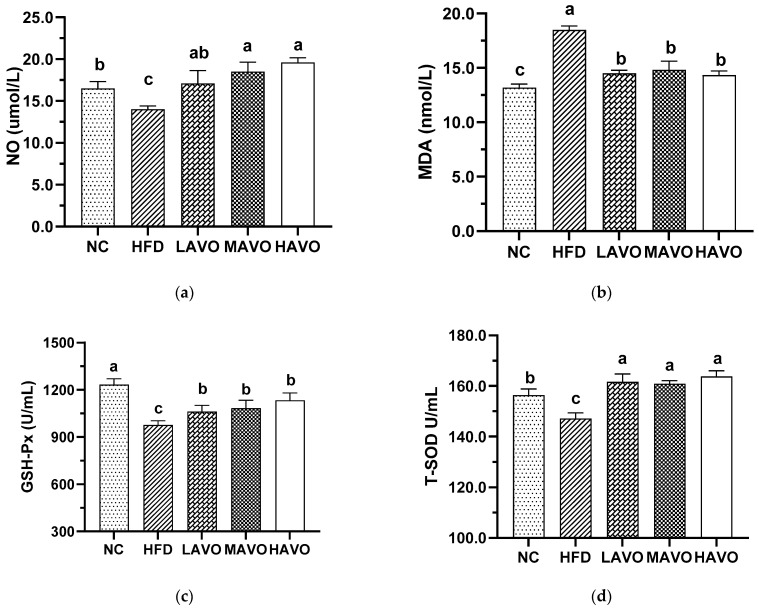
Effect of AVO on the content of NO (**a**), MDA (**b**), GSH-PX (**c**), and T-SOD (**d**) in the mice serum (n = 6). AVO, abalone viscera oil; NC, normal control group; HFD, high-fat diet group; LAVO, high-fat diet + 250 mg/kg AVO; MAVO, high-fat diet + 500 mg/kg AVO; HAVO, high-fat diet + 1000 mg/kg AVO. Different letters indicate statistical significance (*p* < 0.05) between groups.

Oxidative stress, caused by an imbalance in reactive oxygen species (ROS) production, plays a crucial role in the progression of hyperlipidemia [39]. We further measured the serum levels of NO, MDA, GSH-Px, and T-SOD, which are important indicators of oxidative stress. In this study, serum levels of NO, MDA, GSH-Px, and T-SOD in mice fed a high-fat diet were 14.00 μmol/L, 18.48 nmol/mL, 949.96 U/mL, and 147.15 U/mL, respectively (Figure 6). AVO with different-dosage administration significantly increased the content of NO, GSH-Px, and T-SOD to 17.07–19.95 μmol/L, 1077.13–1425.74 U/mL, and 161.00–165.88 U/mL, respectively, and reduced the MDA levels to 14.32–14.50 μmol/L, which was comparable to the levels of the control group (Figure 6). These results suggested that AVO was effective in reducing the level of oxidative stress in hyperlipidemic mice. This was consistent with a previous study that chia seed oil significantly reduces oxidative stress in HFD-induced hyperlipidemic mice [26].

## 4. Discussion

Abalone viscera contain 20.03% of oil content in the dry matter, yet their deep development and comprehensive utilization have not been fully highlighted. In the present study, a multistage countercurrent extraction method was used to extract the oil from abalone viscera. Ferreira et al. reported that a pilot-scale five-stage countercurrent extraction using ethanol as a solvent achieved an extraction rate of 99.2% for soybean oil [14]. Bessa et al. used the same countercurrent extraction method to extract rice bran oil, achieving an extraction rate of 91.87% [10]. However, the extraction rate of abalone viscera oil was 81.18% in this study, which may be attributed to the use of a laboratory-scale four-stage countercurrent extraction method. Although the oil yield was lower than expected, it was still higher than the oil yields obtained using supercritical carbon dioxide from the viscera and skin of Indian mackerel (*Rastrelliger kanagurta*) (9.18% and 38.1%, respectively) [40]. This confirms that multistage countercurrent extraction can achieve reasonable extraction rates for solid substrates.

Acid value, iodine value, and peroxide value are the primary key indicators for assessing the quality and stability of oils [41]. Iodine value and acid value characterize the degree of unsaturation and free acid decomposition of triglyceride oils, respectively, while peroxide value serves as a quality parameter for monitoring lipid oxidation [41]. The iodine and peroxide values of AVO meet the first-grade standard for refined fish oils in the aquaculture industry standards, and the acid value meets the second-grade standards. Consistently, a previous study also showed a slightly higher acid value of 13.73 mg KOH/g for algal oil extracted from the marine macroalgae *Ulva fasciata* [42]. Overall, the refined oil meets the relevant requirements for use in food and food supplements.

The fatty acid composition of abalone is characterized by a high proportion of PUFA, particularly EPA (C20:5n3), DHA (C22:6n3), and ARA (C20:4n6) [43]. The specific fatty acids present and their relative amounts may vary depending on diet, species, and whether the abalone is wild or farmed [43,44,45]. For example, the dominant PUFA in wild abalone is DHA (C22:6n3), whereas in farmed abalone, it is EPA (C20:5n3) [43,44]. Additionally, a higher ARA (C20:4n6) is found in wild abalone [43]. ARA (C20:4n6) and EPA (C20:5n3) were the most abundant PUFAs in two commercially farmed abalone species, *Haliotis discus hannai* Ino and *Haliotis diversicolor*, both in the muscle and viscera [45]. For MUFA, the highest fatty acid is oleic acid (C18:1n9), whether wild, cultured, or raised under various conditions [43,44,45]. Consistently, EPA (C20:5n3) and ARA (C20:4n6) were two of the predominant PUFAs in AVO of the present study, and oleic acid (C18:1n9) was the most dominant MUFA in AVO. However, the fatty acid profiles of edible meat from 13 different seafood species (except for abalone), including tiger prawn, European squid, John dory, bluespotted cornetfish, and Twaite shad, were dominated by DHA (C22:6n3) and EPA (C20:5n3) as the primary unsaturated fatty acids, with DHA (C22:6n3) content higher than EPA (C20:5n3) [46]. Prospective observational studies have confirmed the role of long-chain omega-3 PUFAs—EPA and DHA—in primary prevention of atherosclerotic cardiovascular disease, and their underlying mechanism of action was associated with the lowering of dyslipidemia [2,3]. ARA (C20:4n6) exerts a bidirectional regulatory effect on inflammation. It is primarily metabolized via 5-lipoxygenase to produce pro-inflammatory lipids, such as LTB4, during the early stages of inflammation [47]. In contrast, it promotes the generation of the anti-inflammatory lipid LXA4, mediated by both 5-lipoxygenase and 15-lipoxygenase, in the later stages [47]. The LXA4 contributes to reducing the risk of coronary atherosclerosis. A lower plasma EPA: ARA ratio is correlated with an increased risk of diseases, including coronary artery disease, stroke, myocardial infarction, and vascular disease [48]. However, no conclusive evidence currently exists to substantiate the biological effects of oils rich in ARA and EPA. Moreover, dietary supplementation with long-chain omega-6 PUFA, ARA (C20:4n6), has been shown to effectively improve plasma levels of LDL-C and HDL-C, thereby producing beneficial cardiovascular effects [49]. The substitution of dietary SFAs with MUFA of oleic acid (C18:1n9) has also been depicted to reduce cardiovascular risk by lowering blood lipids, primarily cholesterol [50]. Therefore, AVO is expected to reduce the risk of cardiovascular disease by alleviating dyslipidemia.

Vitamin E and astaxanthin are potent antioxidants naturally present in fish oil, with their concentrations varying depending on the fish species, farming practices, and tissues. The vitamin E content in the liver of *Coregonus peled* fish is 546.30 µg/g, significantly higher than the 127.25 µg/g in fat and 157.43 µg/g in viscera [29]. In Nova Scotian cod liver oil, the content of α-tocopherol ranges from 0.26 to 0.32 mg/g [51]. The astaxanthin content in wild salmon (*Oncorhynchus* species) flesh ranges from 26 to 38 mg/kg, while that in farmed Atlantic salmon is 6 to 8 mg/kg [52]. Moreover, vitamin E and astaxanthin offer numerous health benefits, including antihypertensive, anticancer, and anti-obesity properties due to their antioxidant functions [53,54]. The content of vitamin E and astaxanthin in AVO was 105 mg/kg and 533.8 ± 2.6 mg/kg, respectively, indicating a certain antioxidant property of AVO. The IC_50_ values for the scavenging activity of AVO against DPPH, ABTS, and **·**OH radicals were 6.30 mg/mL, 0.45 mg/mL, and 8.95 mg/mL, respectively. For DPPH radical scavenging activity, an IC_50_ value >150 ppm is considered to indicate weak scavenging activity [55]. *Odonus niger* and cod liver oil exhibited **·**OH radical scavenging activities of 60.06 ± 1.42% and 58.33 ± 1.48%, respectively, at a concentration of 200 μg/mL [56]. However, the IC50 value of AVO for scavenging ABTS free radicals is comparable to that of cod liver oil [35]. Olive oil is widely recognized as an oil possessing potent antioxidant properties, with IC_50_ values for scavenging DPPH, ABTS, and **·**OH free radicals reaching 20.00 μg/mL, 1.21 mg/mL, and 5.33 μg/mL, respectively [57,58]. This suggests a less favorable antioxidant capacity of AVO, which may be related to a lower DHA (C22:6n3) content in AVO than in the oils extracted from fish, including black sea Salmon, Catfish, and Titus [59,60]. However, the content of ARA (C20:4n6), a metabolic precursor of the eicosanoid family of lipid mediators and a rapid and safe regulator of plasma [61], is largely higher than that of the fish oils described above. Marine-derived oils rich in long-chain PUFAs, such as EPA (C20:5n3), DHA (C22:6n3), and ARA (C20:4n6), can reduce oxidative stress by enhancing the body’s antioxidant system and promoting the synthesis of antioxidant enzymes [62,63]. Moreover, AVO contains a certain amount of vitamin E and astaxanthin, both of which contribute to reducing oxidative stress in vivo. Supplementation with astaxanthin can remarkably reduce the biomarkers of oxidative stress in the plasma of overweight and obese Korean adults [64]. Vitamin E can also effectively suppress oxidative stress in patients with polygenic hypercholesterolemia [65]. Therefore, although AVO exhibits unsatisfactory antioxidant activity in vitro, it may possess potent antioxidant effects in vivo.

Oxidative stress occurs when an imbalance between ROS and antioxidant defenses occurs [66]. MDA is a byproduct of lipid peroxidation and is commonly used as a marker to assess the extent of oxidative stress in biological systems [35]. In addition, oxidative stress can trigger the decoupling of endothelial nitric oxide synthase (eNOS), reducing NO synthesis or bioavailability, and exacerbating NO-dependent oxidative stress [67]. The SOD family constitutes the first defense line in the ROS scavenging system, which can convert superoxide anions to hydrogen peroxide in the cytosol, mitochondria, and extracellular matrix [68]. The hydrogen peroxide is converted to water and oxygen in the second line of defense by a redox reaction catalyzed by catalase and GSH [69]. In this study, after 4 weeks of AVO exposure, serum MDA levels were significantly decreased in all dose groups, while NO, GSH-Px, and T-SOD levels increased significantly, indicating reduced oxidative stress in mice. Oxidative stress is an early driver in the development of hyperlipidemia, and providing patients with hyperlipidemia with appropriate antioxidants may help prevent disease progression [66,70]. Typical lipid abnormalities associated with obesity are characterized by elevated TG and total cholesterol TC levels, reduced HDL levels, and abnormal LDL composition [71]. Moreover, elevated cholesterol levels may lead to hepatic lipid accumulation, which can result in liver damage and diseases such as non-alcoholic fatty liver disease (NAFLD) and non-alcoholic steatohepatitis (NASH) [72]. In this study, AVO administration significantly alleviated serum lipid disorders and liver lipid accumulation in HFD-induced mice. The alleviation of oxidative stress is a key mechanism by which long-chain PUFAs, particularly omega-3 fatty acids, can help alleviate dyslipidemia [73]. Astaxanthin and vitamin E are likely to maintain a healthy lipid profile by enhancing oxidative defense mechanisms and reducing inflammatory responses [74,75]. This suggests that AVO may alleviate hyperlipidemia induced by a high-fat diet in mice by reducing oxidative stress.

## 5. Conclusions

Collectively, AVO can be effectively extracted using a multistage countercurrent extraction method, achieving an extraction rate of 81.18%. The iodine, peroxide, and acid values of AVO all meet the standards for refined fish oils specified in the aquaculture industry standards. In AVO, UFAs account for 56.60%, with EPA (C20:5n3) and ARA (C20:4n6) being the two main PUFAs, while oleic acid (C18:1n9) is the most dominant MUFA. AVO also contains a certain amount of vitamin E and astaxanthin, which, together with UFAs, contribute to the antioxidant activity of AVO in vitro. Moreover, supplementation with AVO significantly reduces HFD-induced body weight gain, liver lipid accumulation, and dyslipidemia in mice, which may be related to reduced oxidative stress. These studies suggest that abalone viscera may be a potential source of marine-derived oils for human supplements. 

## 6. Strengths and Limitations

Recent research employed a multistage countercurrent extraction technology using ethanol as a solvent to isolate AVO, achieving high extraction yields. Compared to the traditional extraction solvent hexane, the use of ethanol is safer and more environmentally friendly. This method provides fundamental evidence for the efficient and safe production of AVO. Concurrently, an assessment of the quality and functional characteristics of AVO was conducted, providing a basis for its application in human dietary supplements. However, further research is needed to determine the fatty acid composition of animal liver tissues, the main lipid constituents of the AVO, improve the quality of AVO and its long-term hypolipidemic effects, as well as to elucidate the underlying mechanism of its hypolipidemic action, thereby providing fundamental evidence for the industrialization of AVO production.

## Figures and Tables

**Table 1:** Design of L9 (3^3^) orthogonal array.

	A	B	C	D	
Test Numbers	Solid–Liquid Ratio (g/g)	Extraction Time (mL)	Oscillation Frequency (rpm)	Error Column	cAVO Extraction Rate (%)
1	1 (1:2)	1 (30)	1 (200)	1	69.50
2	1	2 (40)	2 (250)	2	75.34
3	1	3 (50)	3 (300)	3	74.04
4	2 (1:3)	1	2	3	76.24
5	2	2	3	1	81.18
6	2	3	1	2	75.14
7	3 (1:4)	1	3	2	73.09
8	3	2	1	3	78.13
9	3	3	2	1	72.44
K_1_	218.87	218.82	222.77	223.12	∑675.09
K_2_	232.55	234.65	224.01	223.56	
K_3_	223.66	221.62	228.31	228.41	
k_1_	72.94	72.94	74.24	74.39	
k_2_	77.53	78.23	74.69	74.54	
k_3_	74.54	73.89	76.09	76.14	
R	0.91	1.06	0.37	0.35	
The optimal combination	A_2_	B_2_	C_3_		
B > A > C					

**Table 2:** The variance analysis of the orthogonal experiment.

Source of Variance	Sum of Squares	Degree of Freedom	Mean Squares	F Value		Salience
A	1.234	2	0.617	40.490	F_0.01_ (2, 2) = 99.00	*
B	2.061	2	1.031	67.612	F_0.05_ (2, 2) = 19.00	*
C	0.340	2	0.170	11.161		
Error	0.030	2	0.015			

* *p* < 0.05.

**Table 3:** Acid value, iodine value, and peroxide value of cAVO and AVO.

	Aquatic Industry Standard for Fish Oil (SC/T3502-2016) [27]		Standard for Fish Oil CXS 329-2017 [28]	cAVO	AVO
	Refined Fish Oil				
	First Grade	Second Grade			
Acid value (mg KOH/g)	≤1.0	≤3.0	≤3.0	21.73 ± 0.77 a	1.26 ± 0.16 b
Peroxide value (meq/kg)	≤5.0	≤10.0	≤5.0	6.82 ± 0.78 a	3.6 ± 0.32 b
Iodine value (g/100 g)	≥140		-	123.6 ± 2.29 a	140.9 ± 2.84 b

cAVO, crude abalone viscera oil; AVO, abalone viscera oil. Different letters of the row indicate statistical significance (*p* < 0.05) between groups.

**Table 4:** Fatty acid composition of AVO.

Fatty Acid	Relative Content/%	Fatty Acid	Relative Content/%
Lauric acid (C_12:0_)	0.16	Eicosenoic acid (C_20:1n11_)	2.70
Ficocerylic acid (C_13:0_)	0.16	α-Linolenic acid (C_18:3n3_)	0.64
Myristic acid (C_14:0_)	14.47	Heneicosanoic acid (C_21:0_)	0.64
Myristoleic acid (C_14:1n5_)	0.32	Eicosadienoic acid (C_20:2n6_)	1.27
Pentadecanoic acid (C_15:0_)	1.91	8,11,14-Eicosatrienoic acid (C_20:3n6_)	1.11
Palmitic acid (C_16:0_)	23.05	Erucic acid (C_22:1n9_)	0.32
Palmitoleic acid (C_16:1n7_)	4.93	Arachidonic acid (C_20:4n6_)	16.53
Margaric acid (C_17:0_)	0.79	Docosadienoic acid (C_22:2n6_)	0.48
Heptadecenoic acid (C_17:1n7_)	0.64	Eicosapentaenoic acid (C_20:5n3_)	14.63
Stearic acid (C_18:0_)	2.23	Tetracosenic acid (C_24:1n9_)	0.16
Oleic acid (C_18:1n9_)	10.97	SFAs	43.40
Linoleic acid (C_18:2n6c_)	1.59	MUFAs	20.03
γ-Linolenic acid (C_18:3n6_)	0.32	PUFAs	36.57

SFAs, saturated fatty acids; PUFAs, polyunsaturated fatty acids; MUFAs, monounsaturated fatty acids.

## Data Availability

All data generated or analyzed during this study are included in this published article and its Supplementary Information files.

## Supplementary Materials

The following supporting information can be downloaded at: https://www.mdpi.com/article/10.3390/nu17193062/s1, Table S1: The composition of normal chow and high-fat diet. Table S2: The lipid levels in mice fed a high-fat diet and a normal diet for two weeks.

## Abbreviations

The following abbreviations are used in this manuscript:

AVO	abalone viscera oil
UFA	unsaturated fatty acid
SFAs	saturated fatty acids
PUFAs	polyunsaturated fatty acids
MUFAs	monounsaturated fatty acids
TG	triglycerides
TC	total cholesterol
HDL-C	high-density lipoprotein cholesterol
LDL-C	low-density lipoprotein cholesterol
MDA	malondialdehyde
NO	nitric oxide
GSH-Px	glutathione peroxidase
T-SOD	total superoxide dismutase
AC	Atherogenic coefficient
