# Supplementary material for: Multistage Countercurrent Extraction of Abalone Viscera Oil and Its Hypolipidemic Action on High-Fat Diet-Induced Hyperlipidemia Mice
Source: Nutrients. 2025 Sep 25;17(19):3062. doi: 10.3390/nu17193062 (PMC12525568; doi:10.3390/nu17193062)
Supplement: Supplementary file 1 [file nutrients-17-03062-s001.zip › nutrients-3855096-supplementary.pdf]

Table S1. The composition of normal chow and high fat diet

Ingredients (g/kg of diet)	Diets	
	H10010	H10045
Casein	189.58	233.06
L-Cysteine	2.84	3.50
Corn Starch	298.59	84.83
Maltodextrin	33.18	116.53
Sucrose	331.77	201.36
Cellulose	47.40	58.26
Soybean oil	23.70	29.13
Lard	18.96	206.84
Mineral Mix M1002	9.48	11.65
Calcium Phosphate	12.32	15.15
Calcium Carbonate	5.21	6.41
Potassium Citrate	15.64	19.23
Vitamin mix V10001	9.48	11.56
Choline Bitartrate	1.90	2.33
Edible yellow dye	0.047	0
Edible red dye	0	0.058
Total	1000	1000

Table S2. The lipid levels in mice fed a high-fat diet and a normal diet for two weeks

Groups	TG (mmol/L)	TC (mmol/L)	Body weight (g)
NC	0.90 ± 0.13 [a]	3.20 ± 0.19 [a]	37.67 ± 1.15 [a]
HFD	2.11 ± 0.18 [b]	4.25 ± 0.29 [b]	40.31 ± 1.02 [b]

Different letters indicate statistical significance ($P < 0.05$) between groups. NC, normal control group; HFD, high-fat diet group.
